# Supplementary material for: Evaluation of the Approach towards Vaccination against COVID-19 among the Polish Population—In Relation to Sociodemographic Factors and Physical and Mental Health
Source: Vaccines (Basel). 2023 Mar 19;11(3):700. doi: 10.3390/vaccines11030700 (PMC10058603; doi:10.3390/vaccines11030700)
Supplement: Supplementary file 1 [file vaccines-11-00700-s001.zip › vaccines-2235357-supplementary.pdf]

**Table S1:** Questionnaire used in our study to determine the attitude towards influenza and COVID-19 vaccination among Polish respondents (n=200,000).

| **Q1:** Are you vaccinated against influenza? | |
|---|---|
| Points: | Answers: |
| 1 | Yes, every year, including in 2022 |
| 2 | Yes, every year, but I couldn't take an influenza vaccination in 2022 due to the lack of its availability |
| 3 | Yes, but not every year. I got the influenza vaccine in 2022 |
| 4 | Yes, but not every year. I wanted to get a vaccine in 2022, but I couldn't due to the lack of its availability |
| 5 | Usually not, but I got the influenza vaccine in 2022 |
| 6 | Usually not. I wanted to get a vaccine in 2022, but I couldn't due to the lack of its availability. |
| 7 | No, never |
| **Q2:** Are you going to take a vaccine against COVID-19? | |
| 1 | Yes, I have already been vaccinated |
| 2 | Yes, I intend to take a COVID-19 vaccination |
| 3 | I don't know yet |
| 4 | No, never |
| **Q3:** Why won't you take a COVID-19 vaccination? | |
| 1 | I have concerns about the safety of the COVID-19 vaccine |
| 2 | I am afraid of post-vaccination complications |
| 3 | I can't get vaccinated due to medical reasons |
| 4 | I am against vaccination in general |
